# Adiponectin differentially affects gene expression in human mammary epithelial and breast cancer cells

**DOI:** 10.1038/sj.bjc.6604692

**Published:** 2008-09-30

**Authors:** O Treeck, C Lattrich, I Juhasz-Boess, S Buchholz, G Pfeiler, O Ortmann

**Affiliations:** 1 Department of Obstetrics and Gynecology, University of Regensburg, Landshuter Strasse 65, Regensburg 93053, Germany

**Keywords:** adiponectin, mammary epithelial cell, breast cancer cell line, gene regulation

## Abstract

Serum levels of adiponectin are inversely associated with breast cancer risk. In this study, its effect on growth and gene expression of MCF-7 breast cancer cells and MCF-10A human mammary epithelial cells was compared. The antiproliferative effect of adiponectin on MCF-10A cells was more pronounced and was accompanied by elevated transcript levels of caspase 1, ER*β*2, ER*β*5, TR2 and USP2. Our data suggest that upregulation of genes with known growth inhibitory or apoptotic functions in mammary epithelial cells might contribute to the protective action of this adipocytokine.

Adipose tissue acts as an endocrine organ secreting a variety of proteins into the blood known as adipocytokines (Matsuzawa *et al*, 1999). In contrast to other adipocytokines, serum adiponectin concentration is inversely correlated with obesity (Abbasi *et al*, 2004). Low serum adiponectin levels were shown to serve as a significant risk factor for breast cancer, particularly in postmenopausal women (Miyoshi *et al*, 2003).

In this study, we examined the molecular mechanisms underlying the antitumoural action of adiponectin. For this purpose, we studied adiponectin-triggered changes in the oestrogen receptor expression and attempted to identify other genes regulated by this adipocytokine in MCF-7 breast cancer cells and in MCF-10A-immortalised human mammary epithelial cells.

## Materials and methods

### Materials

Full-length low-weight adiponectin expressed in insect H5 cells as recently described (Neumeier *et al*, 2006) was kindly provided by Christa Büchler, Department of Internal Medicine I, University of Regensburg. Serum replacement 2 (SR2) was obtained from Sigma (Deisenhofen, Germany). RNeasy Mini Kit, RNase Free DNase Set and Quantitect SYBR Green PCR Kit were obtained from Qiagen (Hilden, Germany).

### Cell culture and proliferation assay

MCF-7 (HTB-22) breast cancer cells and MCF-10A-immortalised breast epithelial cells (ATCC, Manassas, VA, USA) were maintained in DMEM/F12 medium containing 10% fetal calf serum (FCS), supplemented with 0.01 mg ml^−1^ insulin and 1 mM sodium pyruvate (MCF-7) or with 1 nM epidermal growth factor (EGF) (MCF-10A) and cultured with 5% CO_2_ at 37°C in a humidified incubator. For analysis of adiponectin effects on cell growth, both cell lines were serum starved and cultured in DMEM/F12 containing 1 × SR2. Cells were seeded in 96-well plates in triplicate (2500 cells per well) and were treated with 10 *μ*g ml^−1^ full-length adiponectin. After 0, 3, 4, 5 and 6 days, relative numbers of viable cells were measured in comparison with the untreated control using the fluorimetrical, resazurin-based Cell Titer Blue assay (Promega, Mannheim, Germany) according to the manufacturer's instructions at 560_Ex_/590_Em_ nm in a Victor3 multilabel counter (PerkinElmer, Massachusetts, USA). Cell growth was expressed as a percentage of the untreated medium control. Statistical analysis of the data was performed by one-way ANOVA using Prism 2.0 Software (GraphPad, San Diego, CA, USA), with statistical significance accepted at *P*<0.05.

### RNA preparation and hybridisation of DNA microarrays

Total RNA was isolated by means of the RNeasy kit (Qiagen) according to the manufacturer's instructions. After DNAse treatment and subsequent precipitation, 20 *μ*g of total RNA was used for cDNA synthesis using a cDNA kit (Roche, Mannheim, Germany). After purification of the cDNA, Cy3- and Cy5-labelled cRNA were generated using the MEGAscript™ T7 Kit (Ambion, Woodward, USA). After purification of the labelled cRNA using the High Pure RNA Tissue Kit (Roche) and its fragmentation, 10 *μ*g of Cy3- and Cy5-labelled cRNA were used for the hybridisation of the human 10k-A array (MWG Biotech, Ebersberg, Germany) containing oligonucleotides that represent approximately 10 000 human genes with known functions. The measurement of the slides was performed by the scanning service of MWG Biotech. Data are presented as a relationship between gene expression in untreated MCF-7 or MCF-10A cells (defined as 100%) and the respective expression 48 h after treatment with 10 *μ*g ml^−1^ adiponectin.

### Reverse transcription and real-time PCR

From 1 *μ*g of total RNA, cDNA was synthesised using 100 U M-MLV-P reverse transcriptase (Promega), 2.5 mM dNTP mixture and 50 pM random primers (Invitrogen, Karlsruhe, Germany). All PCR primers were designed intron-spanning to avoid amplification of genomic DNA; primer sequences are indicated in Table 1. For real-time PCR analysis, 4 *μ*l of 1 : 5 diluted cDNA was amplified using the Quantitect SYBR Green PCR Kit (Qiagen) and the LightCycler PCR device (Roche Diagnostics, Mannheim, Germany). The PCR programme was 95°C for 10 min, followed by 40 PCR cycles (95°C for 10 s, 58°C for 10 s, 72°C for 15 s) and a final extension for 5 min at 72°C, followed by a standard melting curve analysis. In all RT–PCR experiments, a 190 bp *β*-actin fragment was amplified as a reference gene using intron-spanning primers. After testing that the efficiencies of all primer pairs were approximately equal (Stahlberg *et al*, 2003), data were analysed using the comparative ΔΔ*C*_T_ method (Livak and Schmittgen, 2001) by calculating the difference between the threshold cycle (*C*_T_) values of the target and reference gene of each sample and then comparing the resulting Δ*C*_T_ values among different samples.

## Results

**Figure 1:**
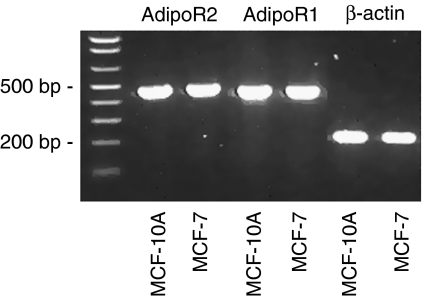
Expression of adiponectin receptors (AdipoR) 1 and 2 in MCF-7 and MCF-10A cells detected by means of RT–PCR. After real-time RT–PCR analysis (35 cycles, LightCycler, Roche), PCR products were subjected to 1.5% agarose gel electrophoresis.

As a pre-requirement for this study, we first tested the expression of adiponectin receptors (AdipoR) 1 and 2 in the cell lines used. By means of real-time RT–PCR, we detected notable amounts of both transcripts in MCF-7 and MCF-10A cells (Figure 1). Receptor mRNA levels were in a similar range in both cell lines (data not shown).

**Figure 2:**
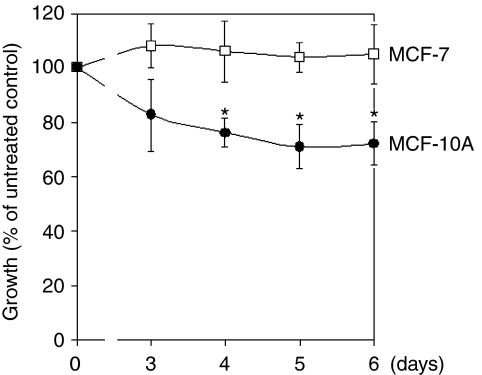
The effect of adiponectin (10 *μ*g ml^−1^) on growth of MCF-7 and MCF-10A cells over a cultivation time of 6 days. Data are expressed in percentage of the untreated control (*n*=3). ^*^P<0.05 *vs* untreated control.

The effect of a physiological concentration of adiponectin on growth of MCF-7 and MCF-10A cells was tested in serum-free, defined culture medium and assessed over a cultivation period of 6 days. Adiponectin significantly inhibited the growth of MCF-10A mammary epithelial cells, but not of MCF-7 breast cancer cells. In relation to the untreated control, this effect was significant after 4 or more days, with a maximum growth reduction by 30% evident after 5 days (Figure 2).

A total of six 10 k DNA microarrays (MWG, Martinsried, Germany) were used to analyse adiponectin effects on gene expression. Total RNA was isolated from MCF-7 and MCF-10A cells cultured in serum-free, defined culture medium treated with adiponectin (10 *μ*g ml^−1^) for 48 h. Microarray data confirmed by 2-fold repetition of the experiment including dye swap revealed a more than 5-fold increase of transcript levels of caspase 1 (CASP1) (5.94-fold), TR2 orphan nuclear hormone receptor (6.31-fold) and ubiquitin-specific peptidase 2 (USP2) (5.37-fold) in MCF-10A cells. These effects were not observed in microarray experiments comparing the transcriptome of MCF-7 cells before and after adiponectin treatment (Table 2).

**Figure 3:**
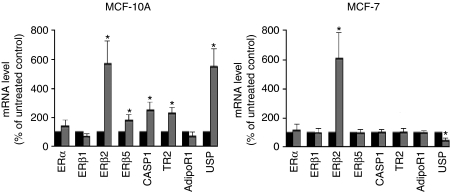
RT–PCR analysis of the adiponectin effects on gene expression in MCF-10A mammary epithelial cells and MCF-7 breast cancer cells. Grey bars represent gene expression after 48 h of treatment with adiponectin (10 *μ*g ml^−1^). Data are expressed in percentage of untreated control (black bars, defined as 100%). ^*^P<0.05 *vs* untreated control.

More than 5-fold expression changes after adiponectin treatment observed in the microarray experiments in case of the genes CASP1, TR2 and USP2 were now confirmed by means of RT–PCR. Increased levels of these genes in adiponectin-treated MCF-10A cells were also detected by means of real-time RT–PCR, although this increase was only 2.5-fold in case of TR2 and USP2 (Figure 3). Given that our recent results suggested interactions between oestrogen receptor (ER) and adiponectin signalling (Pfeiler *et al*, 2008), adiponectin effects on expression of ER*α* and ER*β*-isoforms were also examined in these RT–PCR experiments. In MCF-10A cells, we observed a more than 5-fold increase of ER*β*2 and an approximately 2-fold increase of ER*β*5 mRNA levels after adiponectin treatment. As expected from our microarray data, adiponectin did not trigger elevated transcript levels of TR2, CASP1 and USP2 in MCF-7 breast cancer cells, but a significantly reduced USP2 expression by approximately 60%. Similar to the mammary epithelial cell line, in MCF-7 cells ER*β*2 mRNA levels were also upregulated by more than 5-fold after adiponectin treatment.

## Discussion

Adiponectin levels are inversely related to the prognostic factors of breast cancer (Miyoshi *et al*, 2003; Mantzoros *et al*, 2004). Furthermore, direct antitumoural effects of adiponectin such as inhibition of proliferation have been reported from *in vitro* studies (Kang *et al*, 2005, 2007; Nakayama *et al*, 2007). These observations suggested that adiponectin might be able to protect from breast cancer development. In this study, we examined the effects of adiponectin on proliferation and gene expression of immortalised mammary epithelial cells in comparison with breast cancer cells.

Recently, expression of both AdipoR was reported to be present in various benign mammary epithelial and breast cancer cell lines (Takahata *et al*, 2007). In this study, mRNA levels of AdipoR1 and AdipoR2 were shown to be expressed in a similar range in MCF-7 and MCF-10A cells. However, treatment with adiponectin exerted different effects on these cell lines. Physiological concentrations of this adipocytokine significantly slowed down the proliferation of MCF-10A mammary epithelial cells, but not of MCF-7 breast cancer cells. The results of earlier studies examining the effect of adiponectin on MCF-7 breast cancer cells are contradictory. Our group and others had earlier reported the absence of adiponectin effects on the growth of MCF-7 breast cancer cells (Kang *et al*, 2005; Pfeiler *et al*, 2008). Although some studies reported small apoptotic effects of adiponectin on this cell line not significantly affecting the total cell numbers (Arditi *et al*, 2007; Grossmann *et al*, 2008), in another study, growth-inhibitory effects on MCF-7 cells were observed (Dieudonne *et al*, 2006). However, this inconsistency might result from different experimental conditions used in the studies mentioned. In an earlier study, an antiproliferative effect of adiponectin on MCF-7 cells was not present under serum-free culture conditions as used in this study and was suggested to be dependent on the presence of steroid hormones such as 17-*β* estradiol (Pfeiler *et al*, 2008).

To our knowledge, this is the first study demonstrating a significant growth inhibitory effect of adiponectin on mammary epithelial cells. This observation might reveal at least one mechanism underlying the association between high adiponectin serum levels and reduced breast cancer risk. It is tempting to speculate that adiponectin might be able to exert protective effects on mammary epithelial cells. The lack of any antiproliferative effect on MCF-7 breast cancer cells suggests that adiponectin-triggered growth control might be lost during carcinogenesis. Given that MCF-7 cells expressed AdipoR to a similar extent, the molecular mechanisms of this different response remained to be elucidated.

Given that earlier data of our group suggested the presence of a cross talk between adiponectin and oestrogen signalling systems (Pfeiler *et al*, 2008), we first analysed the effect of an adiponectin treatment on ER*α* and *β* expression. In MCF-10A cells, adiponectin triggered a significant increase of ER*β*2 and ER*β*5 mRNA levels. ER*β* is the dominant oestrogen receptor in normal breast tissue (Speirs *et al*, 2000, 2002; Shaw *et al*, 2002), but its expression declines during tumorigenesis (Leygue *et al*, 1998; Roger *et al*, 2001). This study and a variety of *in vitro* studies of our group and others suggested a role for ER*β* as a tumour suppressor in breast tissue (Skliris *et al*, 2003; Treeck *et al*, 2008). ER*β*2 and ER*β*5 proteins both are known to form heterodimers with ER*α*, thereby inhibiting the transcriptional activity of ER*α* (Peng *et al*, 2003). As ER*α* is known to mediate tumour-promoting effects of 17-*β* estradiol, our data suggest that adiponectin-triggered increase of ER*β* isoform expression might be one important molecular mechanism underlying the protective effects of this adipocytokine. This seems to be particularly true for ER*β*5 splice variant, as the expression of this isoform was exclusively increased in MCF-10A cells.

Then, we studied the gene expression changes triggered by adiponectin in MCF-7 and MCF-10A cells by means of DNA microarrays. After treatment with adiponectin, in MCF-10A mammary epithelial cells, we observed elevated transcript levels of several genes known to exert antitumoural effects in breast tissue.

Adiponectin treatment led to increased transcript levels of TR2 orphan nuclear hormone receptor in MCF-10A, but not in MCF-7 cells. Recent results of *in vitro* studies of cultured cells ectopically expressing TR2 suggested potential roles for TR2 in a variety of cellular processes (Park *et al*, 2007). Interestingly, the transcriptional activity of ER*α*, leading to enhanced proliferation of breast epithelial cells and to tumour progression, can be inhibited not only by ER*β*, but also by TR2. Furthermore, TR2-siRNA was reported to enhance ER*α*-dependent proliferation of MCF-7 cells (Hu *et al*, 2002). Our data suggest that adiponectin-triggered increase of TR2 expression could also lead to the suppression of ER*α*-mediated proliferation in mammary epithelial cells, thereby exerting protective, antitumoural effects.

TR2 has also been reported to be upregulated during apoptotic processes and its overexpression resulted in an increased apoptosis rate (Lee and Wei, 2000). It is tempting to speculate that adiponectin-triggered increase of TR2 might be able to confer an increased apoptotic potential to mammary epithelial cells, thereby also exerting protective effects.

In MCF-10A mammary epithelial cells treated with adiponectin, we also found a significant increase of USP2 mRNA levels, but a decrease in MCF-7 breast cancer cells. In breast cancer, an enhanced activity of the ubiquitin–proteasome system has been reported (Deng *et al*, 2007). However, the exact role of USP in breast tissue remains unknown. Ubiquitination may be a candidate for either a tumour-suppressive or an oncogenic activity (Fujiwara *et al*, 1998).

Adiponectin treatment of MCF-10A cells also resulted in elevated transcript levels of CASP1 , a key enzyme of apoptosis. This effect again was not observed in MCF-7 breast cancer cells. Our data suggest that adiponectin-triggered increase of CASP1 expression might be another molecular mechanism underlying the antitumoural effect of this adipocytokine, which is present in mammary epithelial cells, but has been lost in MCF-7 breast cancer cells. Adiponectin-triggered activation of caspase expression has also been reported from endothelial cells, resulting in decreased angiogenesis (Brakenhielm *et al*, 2004). Given that decreased neovascularisation exerts antitumoural effects, the antiangiogenetic effect of this adipocytokine mediated by CASP1 upregulation might be another mechanism underlying the inverse association between adiponectin serum levels and breast cancer risk.

In this study, we observed an adiponectin-triggered growth inhibition of MCF-10A cells accompanied by specific upregulation of genes with known antitumoural properties. Our data suggest that adiponectin might be able to exert protective effects on mammary epithelial cells only, whereas MCF-7 breast cancer cells might have lost the ability to respond to this adipocytokine in a similar manner.

## Figures and Tables

**Table 1:** Sequences of the PCR primers used in this study

AdipoR1	5′-GGGGAATTCTCTTCCCACAAAGGATCTGTGGTG-3′
	5′-GGGCTGCAGTTAAGTTTCTGTATGAATGCGGAAGAT-3′
AdipoR2	5′-GGGGAATTCAACGAGCCAACAGAAAACCGATTG-3′
	5′-GGGCTGCAGCTAAATGTTGCCTGTTTCTGTGTGTAT-3′
ERα	5′-TGATGAAAGGTGGGATACGA-3′
	5′-AAGGTTGGCAGCTCTCATGT-3′
ERβ1	5′-TTTGGGTGATTGCCAAGAGC-3′
	5′-AGCACGTGGGCATTCAGC-3′
ERβ2	5′-CCCAGAGGGAAACTGAAGTG-3′
	5′-AACCTCCTGATGCTCCTGTC-3′
ERβ5	5′-CACTTTTCCCAAATCACTTCACC-3′
	5′-TTTGGGTGATTGCCAAGAGC-3′
USP2	5′-CATCCAGAGGTTCCCAAAGA-3′
	5′-CCAGGTCTCTTAGGGGGAAG-3′
CASP1	5′-TCTTCCTTTCCAGCTCCTCA-3′
	5′-CTGCCGACTTTTGTTTCCAT-3′
TR2	5′-CACAACACCTGCAGCTCCTA-3′
	5′-TGACGTCCTGATGCTTTGTC-3′
β-actin	5′-CTGTGGCATCCACGAAACTA-3′
	5′-CGCTCAGGAGGAGCAATG-3′

**Table 2:** Gene expression changes detected by means of microarray analysis (10 k-A array, MWG Biotech, Martinsried, Germany) in MCF-7 breast cancer cells and MCF-10A mammary epithelial cells after treatment with adiponectin (NS=not significant) (*n*=3 including dye swap)

	**MCF-10A**	**MCF-7**
**GENE**	**Factor**	**Factor**
TR2	6.31	NS
CASP1	5.94	NS
USP2	5.37	NS
ZNF235	4.49	NS
SERPINB13	3.67	NS
MGST2	3.40	NS
RNF130	3.28	NS
SLC25A20	0.22	NS
DDX59	NS	4.17
GIMAP5	NS	3.64
NRG1	NS	3.22
PSCD3	NS	0.27

Microarray analysis based on ANOVA was performed and the cut off value was a 3-fold difference.

## References

[bib1] Abbasi F, Chu J, Lamendola C, McLaughlin T, Hayden J, Reaven G, Reaven P (2004) Discrimination Between Obesity and Insulin Resistance in the Relationship With Adiponectin. Diabetes 53: 585–590 14988241 10.2337/diabetes.53.3.585

[bib2] Arditi JD, Venihaki M, Karalis KP, Chrousos GP (2007) Antiproliferative effect of adiponectin on MCF7 breast cancer cells: a potential hormonal link between obesity and cancer. Horm Metab Res 39: 9–13 17226107 10.1055/s-2007-956518

[bib3] Brakenhielm E, Veitonmaki N, Cao R, Kihara S, Matsuzawa Y, Zhivotovsky B, Funahashi T, Cao Y (2004) Adiponectin-induced antiangiogenesis and antitumor activity involve caspase-mediated endothelial cell apoptosis. Proc Natl Acad Sci USA 101: 2476–2481 14983034 10.1073/pnas.0308671100 PMC356975

[bib4] Deng S, Zhou H, Xiong R, Lu Y, Yan D, Xing T, Dong L, Tang E, Yang H (2007) Over-expression of genes and proteins of ubiquitin specific peptidases (USPs) and proteasome subunits (PSs) in breast cancer tissue observed by the methods of RFDD-PCR and proteomics. Breast Cancer Res Treat 104: 21–30 17004105 10.1007/s10549-006-9393-7

[bib5] Dieudonne MN, Bussiere M, Dos Santos E, Leneveu MC, Giudicelli Y, Pecquery R (2006) Adiponectin mediates antiproliferative and apoptotic responses in human MCF7 breast cancer cells. Biochem Biophys Res Commun 345: 271–279 16678125 10.1016/j.bbrc.2006.04.076

[bib6] Fujiwara T, Saito A, Suzuki M, Shinomiya H, Suzuki T, Takahashi E, Tanigami A, Ichiyama A, Chung CH, Nakamura Y, Tanaka K (1998) Identification and chromosomal assignment of USP1, a novel gene encoding a human ubiquitin-specific protease. Genomics 54: 155–158 9806842 10.1006/geno.1998.5554

[bib7] Grossmann ME, Nkhata KJ, Mizuno NK, Ray A, Cleary MP (2008) Effects of adiponectin on breast cancer cell growth and signaling. Br J Cancer 98: 370–379 18182989 10.1038/sj.bjc.6604166 PMC2361440

[bib8] Hu YC, Shyr CR, Che W, Mu XM, Kim E, Chang C (2002) Suppression of estrogen receptor-mediated transcription and cell growth by interaction with TR2 orphan receptor. J Biol Chem 277: 33571–33579 12093804 10.1074/jbc.M203531200

[bib9] Kang JH, Lee YY, Yu BY, Yang BS, Cho KH, Yoon DK, Roh YK (2005) Adiponectin induces growth arrest and apoptosis of MDA-MB-231 breast cancer cell. Arch Pharm Res 28: 1263–1269 16350853 10.1007/BF02978210

[bib10] Kang JH, Yu BY, Youn DS (2007) Relationship of serum adiponectin and resistin levels with breast cancer risk. J Korean Med Sci 22: 117–121 17297263 10.3346/jkms.2007.22.1.117 PMC2693546

[bib11] Lee CH, Wei LN (2000) Characterization of the mouse nuclear orphan receptor TR2-11 gene promoter and its potential role in retinoic acid-induced P19 apoptosis. Biochem Pharmacol 60: 127–136 10807954 10.1016/s0006-2952(00)00311-7

[bib12] Leygue E, Dotzlaw H, Watson PH, Murphy LC (1998) Altered estrogen receptor alpha and beta messenger RNA expression during human breast tumorigenesis. Cancer Res 58: 3197–3201 9699641

[bib13] Livak KJ, Schmittgen TD (2001) Analysis of relative gene expression data using real-time quantitative PCR and the 2(-Delta Delta C(T)) Method. Methods 25: 402–408 11846609 10.1006/meth.2001.1262

[bib14] Mantzoros C, Petridou E, Dessypris N, Chavelas C, Dalamaga M, Alexe D, Papadiamantis Y, Markopoulos C, Spanos E, Chrousos G, Trichopoulos D (2004) Adiponectin and breast cancer risk. J Clin Endocrinol Metab 89: 1102–1107 15001594 10.1210/jc.2003-031804

[bib15] Matsuzawa Y, Funahashi T, Nakamura T (1999) Molecular mechanism of metabolic syndrome X: contribution of adipocytokines adipocyte-derived bioactive substances. Ann N Y Acad Sci 892: 146–154 10842660 10.1111/j.1749-6632.1999.tb07793.x

[bib16] Miyoshi Y, Funahashi T, Kihara S, Taguchi T, Tamaki Y, Matsuzawa Y, Noguchi S (2003) Association of serum adiponectin levels with breast cancer risk. Clin Cancer Res 9: 5699–5704 14654554

[bib17] Nakayama S, Miyoshi Y, Ishihara H, Noguchi S (2007) Growth-inhibitory effect of adiponectin via adiponectin receptor 1 on human breast cancer cells through inhibition of S-phase entry without inducing apoptosis. Breast Cancer Res Treat [Epub ahead of print] 10.1007/s10549-007-9874-3 18163210

[bib18] Neumeier M, Weigert J, Schaffler A, Wehrwein G, Muller-Ladner U, Scholmerich J, Wrede C, Buechler C (2006) Different effects of adiponectin isoforms in human monocytic cells. J Leukoc Biol 79: 803–808 16434692 10.1189/jlb.0905521

[bib19] Park SW, Hu X, Gupta P, Lin YP, Ha SG, Wei LN (2007) SUMOylation of Tr2 orphan receptor involves Pml and fine-tunes Oct4 expression in stem cells. Nat Struct Mol Biol 14: 68–75 17187077 10.1038/nsmb1185

[bib20] Peng B, Lu B, Leygue E, Murphy LC (2003) Putative functional characteristics of human estrogen receptor-beta isoforms. J Mol Endocrinol 30: 13–29 12580758 10.1677/jme.0.0300013

[bib21] Pfeiler GH, Buechler C, Neumeier M, Schäffler A, Schmitz G, Ortmann O, Treeck O (2008) Adiponectin effects on human breast cancer cells are dependent on 17-beta estradiol. Oncol Rep 19: 787–793 18288417

[bib22] Roger P, Sahla ME, Makela S, Gustafsson JA, Baldet P, Rochefort H (2001) Decreased expression of estrogen receptor beta protein in proliferative preinvasive mammary tumors. Cancer Res 61: 2537–2541 11289127

[bib23] Shaw JA, Udokang K, Mosquera JM, Chauhan H, Jones JL, Walker RA (2002) Oestrogen receptors alpha and beta differ in normal human breast and breast carcinomas. J Pathol 198: 450–457 12434414 10.1002/path.1230

[bib24] Skliris GP, Munot K, Bell SM, Carder PJ, Lane S, Horgan K, Lansdown MR, Parkes AT, Hanby AM, Markham AF, Speirs V (2003) Reduced expression of oestrogen receptor beta in invasive breast cancer and its re-expression using DNA methyl transferase inhibitors in a cell line model. J Pathol 201: 213–220 14517838 10.1002/path.1436

[bib25] Speirs V, Adams IP, Walton DS, Atkin SL (2000) Identification of wild-type and exon 5 deletion variants of estrogen receptor beta in normal human mammary gland. J Clin Endocrinol Metab 85: 1601–1605 10770204 10.1210/jcem.85.4.6493

[bib26] Speirs V, Skliris GP, Burdall SE, Carder PJ (2002) Distinct expression patterns of ER alpha and ER beta in normal human mammary gland. J Clin Pathol 55: 371–374 11986344 10.1136/jcp.55.5.371 PMC1769648

[bib27] Stahlberg A, Aman P, Ridell B, Mostad P, Kubista M (2003) Quantitative real-time PCR method for detection of B-lymphocyte monoclonality by comparison of kappa and lambda immunoglobulin light chain expression. Clin Chem 49: 51–59 12507960 10.1373/49.1.51

[bib28] Takahata C, Miyoshi Y, Irahara N, Taguchi T, Tamaki Y, Noguchi S (2007) Demonstration of adiponectin receptors 1 and 2 mRNA expression in human breast cancer cells. Cancer Lett 250: 229–236 17123704 10.1016/j.canlet.2006.10.006

[bib29] Treeck O, Juhasz-Boess I, Lattrich C, Horn F, Goerse R, Ortmann O (2008) Effects of exon-deleted estrogen receptor beta transcript variants on growth, apoptosis and gene expression of human breast cancer cell lines. Breast Cancer Res Treat 110: 507–520 17876701 10.1007/s10549-007-9749-7

